# Pinyin Spelling Promotes Reading Abilities of Adolescents Learning Chinese as a Foreign Language: Evidence From Mediation Models

**DOI:** 10.3389/fpsyg.2020.596680

**Published:** 2020-12-09

**Authors:** Huimin Xiao, Caihua Xu, Hetty Rusamy

**Affiliations:** ^1^Chinese Language and Culture College, Beijing Normal University, Beijing, China; ^2^Chinese Department, Xin Zhong School, Surabaya, Indonesia

**Keywords:** Pinyin, Chinese, Pinyin spelling, Chinese reading abilities, Chinese as a foreign language (CFL)

## Abstract

Pinyin is a phonological encoding system used to spell modern Chinese Mandarin due to the phonological opacity of Chinese characters. The present study examined the role of Pinyin spelling in the reading abilities of adolescents learning Chinese as a foreign language (CFL). A total of 158 Indonesian senior primary students were tested on Pinyin spelling, character production, listening comprehension, depth of vocabulary knowledge, and reading comprehension. Pinyin spelling skills were assessed by two measures, Pinyin Dictation (sentence dictation in Pinyin) and Pinyin Tagging (Pinyin writing for characters). Path analysis revealed that even after controlling for the effect of character production, Pinyin dictation performance influenced reading comprehension through the mediating effect of listening comprehension and the depth of vocabulary knowledge, and Pinyin tagging performance also influenced reading comprehension through the mediating effect of the depth of vocabulary knowledge. The results highlight the importance of Pinyin skills for Chinese reading abilities of CFL learners. As a reliable and explicit indicator of specifying Chinese phonological representations and processing, Pinyin spelling has a long-term and multifaceted influence on higher-level CFL abilities.

## Introduction

Chinese characters, functioning as the written symbol of Chinese language, are famous for their phonological opacity as a morphosyllabic visual script that is significantly different from alphabetic scripts (Mattingly, 1992; Hanley, 2005). Therefore, Pinyin, an alphabetic encoding system, was invented in 1956 in Mainland China to encode the phonological system of Mandarin Chinese and has become extremely important in the process of learning the Chinese writing system. Native Chinese children and nonnative Chinese learners rely on Pinyin to pronounce unfamiliar characters and establish print-sound correspondence (Shu and Liu, 1994; Zhang et al., 2020). Studies have shown that Pinyin letter knowledge or Pinyin spelling is beneficial for Chinese character reading in native Chinese children (Siok and Fletcher, 2001; Lin et al., 2010; Yin et al., 2011; Wang et al., 2014; Wang and McBride, 2016; Ding et al., 2018; Ma et al., 2020; Zhang et al., 2020). However, there are few studies of the role of Pinyin spelling skills in Chinese as a foreign language (CFL) or Chinese as a second language (CSL) learners’ development of Chinese reading, let alone the mechanism of influence and whether the influence is limited to primary learners. Therefore, the present study aims to fill this gap by examining if Pinyin spelling could influence the reading abilities of senior primary CFL learners beyond the influence of known Chinese character production.

### Chinese Pinyin

Pinyin (formally known as Hanyu Pinyin) is an alphabetic encoding system that spells modern Chinese Mandarin according to the phonemic principle using internationally accepted Roman letters (Scheme of the Chinese Phonetic Alphabet, 1958). The Pinyin system employs 21 onsets and 39 rimes composed of 26 letters, plus four lexical tones to transcribe the pronunciation of all Chinese characters (Institute of Linguistics, Chinese Academy of Social Sciences, 2004). The Pinyin letter names correspond perfectly to the sounds they represent. However, simply mastering the 26 letters is not enough to spell Pinyin syllables, and only by mastering the unique representations of onsets, rimes and tones, and the rules of spelling can learners truly understand the characteristics of Chinese phonology. The structure of Chinese syllables is relatively simple with strong regularity and the tone is important; both structure and tone are well reflected in Pinyin encoding system.

At present, CFL/CSL beginners typically spend 1–2 weeks to learn Pinyin systematically in the case of more Chinese teaching time per week. In addition, all the pronunciations of new words are explicitly printed with Pinyin in the Chinese textbooks of learners, even those with high Chinese proficiency, and CFL/CSL teachers generally use Pinyin to correct students’ pronunciation in spoken language. In contrast, Pinyin is much more important in overseas Chinese teaching with fewer class hours and has played a leading role in children’s CFL learning for quite a long time. Nevertheless, the best transition time and method from Pinyin to Chinese character teaching has not been determined yet, and there is still skepticism regarding Pinyin instruction (Xu and Liu, 2016). For example, some people think that Pinyin may interfere with the acquisition of native words in children with an alphabet language as a first language (L1), or they can spell Pinyin without learning.

Researchers in the field of CFL/CSL teaching attach great importance to the role of Pinyin in Chinese teaching, and believe that Pinyin has at least the following four functions: (a) Pinyin helps to bridge the gap between phonology and orthography in Chinese reliably and directly, which is particularly useful given the unreliability of phonetic radicals within Chinese characters; (b) Pinyin enables learners to smoothly acquire the Chinese phonological system avoiding the difficulties in writing Chinese characters and learning to speak Chinese, and to gain a sense of achievement in communication earlier; (c) The rules of word segmentation and serial writing in spelling Pinyin sentences can help learners to segment words and understand the mixed use of single and two-syllable words in Chinese; and (d) Pinyin has the convenience of stenography and computer input (Pinyin Typewriting), which allows Pinyin to surpass the status of a teaching aid and play the role of communicative scripts, etc. (Chung, 2002; Wan, 2012; Lu, 2013; Wang, 2013; Xu and Liu, 2016).

### Pinyin Spelling and Chinese Phonological Representations

Pinyin skills are usually assessed by several measures, including Pinyin letter knowledge, invented Pinyin spelling and Pinyin spelling. To evaluate Pinyin knowledge, tasks are commonly administered that require learners to recognize Pinyin letters or read Pinyin syllables/sentences quickly (Yin et al., 2011; Wang et al., 2014; Wang and McBride, 2016; Zhang et al., 2020). Invented Pinyin spelling is adapted from invented spelling in English (Read, 1971; Bryant and Bradley, 1980) and places particular emphasis on the ability to spontaneously develop Chinese phonological acuity, especially in preschool Chinese children who have not been introduced to Pinyin formally (Lin et al., 2010; Pan et al., 2011). For CFL/CSL learners, who have received Pinyin instruction systematically, Pinyin spelling has been explored as two specific tasks in the present study, Pinyin Dictation (Sentence diction in Pinyin) and Pinyin Tagging (Pinyin writing for characters).

Compared with Pinyin letter knowledge, Pinyin spelling is a much more complicated task because success in this domain demands establishing fine-grained Chinese phonological representations. Pinyin letter knowledge has a dramatic developmental trajectory, and CFL/CSL learners’ Pinyin proficiency generally reaches a relatively high level within a short period (According to our pilot test, CFL learners’ of Pinyin knowledge is 80–90 percent accurate). However, considering that speech learning in a second language (L2) is affected by some elaborate perceptual similarities and dissimilarities between native and nonnative phonemes (Flege, 1999; Best and Tyler, 2007), there are still some universal difficulties with specific segments and contrasts across different L1s in Chinese phonological acquisition, such as coronal affricates and fricatives, including [tɕ tɕ^h^ ɕ], [ts ts^h^ s], and [tʂ tʂ^h^ ʂ], represented as letters: *j q x z c s zh ch sh* in the Pinyin alphabet. In the three groups of consonants, the manner of articulation between /z c s/ and /zh ch sh/ corresponds one to one but varying in places of articulation (apico-alveolar vs. post-alveolar). In addition, /j q x/ and /zh ch sh/ are the same in manner but differ in adjacent places of articulation (alveolar-palatal vs. post-alveolar; Cheng and Zhao, 1985; Deng, 2018). Consequently, higher Pinyin spelling performance on these related difficult Pinyin words might function as a reliable and explicit indicator of the successful categorization of these consonants, which means that CFL/CSL learners can capture the discrimination of these different phonemes and achieve fine-grained Chinese phonological representations.

Furthermore, Pinyin spelling reflects the essential role of Pinyin in CFL/CSL learning, using Pinyin representations to phonologically encode spoken Chinese and transcode Chinese characters in Pinyin forms. Correspondingly, the measures of Pinyin spelling in the present study have taken into account both cognitive functions of Pinyin. Sentence dictation in Pinyin relates to encoding Chinese speech information in Pinyin, and writing out corresponding Pinyin according to Chinese characters involves transcoding character orthography to Pinyin forms. Pinyin spelling can assess the learners’ basic knowledge of Pinyin letters and the rules of spelling together onset, rime, and tone because the end-result of both tasks is the spelling of Pinyin symbols. More importantly, the former can also examine CFL/CSL learners’ discriminability, sensitivity, and accuracy of mental representations of phonetic categories, while the latter can also capture a learners’ association degree between orthography, phonology and semantics when identifying Chinese words.

Some studies suggested that compared with traditional measures (e.g., Syllable Deletion and Phoneme Deletion), Pinyin spelling or invented spelling may be an even better measure and proxy for Chinese phonological awareness (Lin et al., 2010; Ding et al., 2018). First, Pinyin spelling underscores using a phonics approach to manipulate sounds, including segmenting a syllable into onset, rime, tone and phonemes, and correspondingly, to reconnect them into a whole syllable. Second, Pinyin spelling better captures the unique linguistic features of Chinese that do not exist in Indo-European alphabetic languages, including tone awareness, tone sandhi, and a complete phonological structure in order (onset, rime, and tone). In alphabetic languages, spelling skills promote subsequent word decoding and reading (Bradley, 1988; Ehri, 1989; Ouellette and Sénéchal, 2008), and both orthographic and phonological processes are required, as their codes are overlapping (McBride-Chang, 1998). However, the Chinese orthographic and phonological codes are different. Do the phonological codes, namely, Pinyin, spelling also affect Chinese reading ability when Pinyin does not constitute basic units of reading in Chinese itself?

### Pinyin Spelling in Relation to CFL Reading Comprehension

Reading comprehension is the ultimate goal of reading instruction (Snow, 2002), and thus a suitable task to investigate the deep influence of Pinyin spelling on nonprimary learners’ reading abilities. According to the simple view of reading (SVR, Hoover and Gough, 1990), reading comprehension is the product of word decoding and listening comprehension, which emphasizes the role of speech decoding of written words and oral comprehension in word recognition and higher-level cognitive processing. The model has been extensively verified in research on languages differing in orthographic depth, and both L1 and L2 readers (Florit and Cain, 2011; Janssen et al., 2017), including CSL learners (Wong, 2016). Beyond SVR, recent research has highlighted the importance of the quality of word representations for reading skill, including comprehension (lexical quality hypothesis, LQH, Perfetti and Hart, 2001; Droop and Verhoeven, 2003; Perfetti, 2007; Perfetti and Stafura, 2014; Verhoeven et al., 2019). Relevant scholars find that mastering and specifying the knowledge of word constituents (orthography, phonology, morpho-syntax, and semantics) and the constituent bond help identify the word in the mental lexicon, and in turn free more mental resources to understand, integrate, and infer the meaning of the input words.

Theoretically, Pinyin spelling skills might effectively facilitate Chinese reading comprehension *via* at least two pathways. First, listening comprehension may mediate the effects of Pinyin sentence dictation on reading comprehension of CFL/CSL learners, because the Pinyin encoding process can indicate the mastery of the Chinese phonological system, and foster the perception and analytical skills of the Chinese oral language by using explicit Pinyin representations. Accordingly, Pinyin encoding skill might be closely related to learners’ listening comprehension performance, and then impacts on reading comprehension. However, we assumed that Pinyin writing for characters might not predict listening comprehension directly.

Second, the depth of vocabulary knowledge may mediate the relationship between Pinyin spelling and Chinese reading comprehension. Depth of vocabulary knowledge refers to how well a learner knows a word (Read, 1993), and Nation (2001) proposed three facets of word knowledge: form (including spoken form and written form), meaning, and use (incorporating grammatical functions, collocations and constraints on use). To some degree, the learned mapping between Pinyin and Chinese character arise from learners’ Pinyin experiences, and compared with native Mandarin speakers, CFL/CSL learners rely more on Pinyin to pronounce characters and memorize the orthography, phonology, and meaning simultaneously. Pinyin encoding can strengthen the stability and accessibility of the phonological representations of words, and higher transcoding skill also implies the flexible and accurate extraction of phonological information from even homophonic, polyphonic, and similar characters. Both skills may directly predict the depth of vocabulary knowledge, which in turn predicts reading comprehension.

### The Present Study

In the present study, we examined the influence of Pinyin spelling on reading abilities in a sample of senior primary CFL students. We expected that Pinyin spelling resulted in cognitive functions that could contribute to their reading abilities, even reading comprehension, of high-grade pupils without ongoing intensive Pinyin training. Specifically, our first hypothesis was that listening comprehension would mediate the effects of Sentence dictation in Pinyin on reading comprehension, and our second hypothesis was that the depth of vocabulary knowledge would mediate the effect of Pinyin spelling abilities (both Sentence dictation in Pinyin and Pinyin writing for characters) on reading comprehension.

Although there are opposing studies (e.g., Bi et al., 2009), some studies suggested that reading in Chinese depends on character writing (Tan et al., 2005; Wang et al., 2014). Hence, we measured learners’ competence of producing Chinese characters as a control variable. The task of character production (character dictation) can assess learners’ ability to access and produce the orthographic representation through phonological input, which can be further compared with the effects of the two Pinyin spelling tasks, especially sentence dictation in Pinyin.

The present study is important for both theoretical and practical reasons. From a theoretical point of view, there have been few studies to date of the cognitive functions of Pinyin in CFL/CSL acquisition. This paper explored the influence of Pinyin on reading comprehension, making clear the similarities and differences between the influence mechanisms of Pinyin spelling and character production, and can inform a series of related issues, such as the role of Pinyin (including onsets, rimes, and tones) for promoting oral Chinese and literacy acquisition in CFL/CSL learners. From a practical perspective, our findings will provide insights for whether teachers should teach Pinyin and to what extent Pinyin should be taught in overseas Chinese teaching environments, as well as a series of specific practical questions such as whether Pinyin learning or practice should appear exclusively at the initial stage of Chinese learning.

## Materials and Methods

### Participants

The participants were 158 senior primary students from an International School in Surabaya, Indonesia (71 male, 87 female; mean age = 11.79, *SD* = 1.44). All participants had passed the Youth Chinese Test (YCT) 4 with intermediate Chinese proficiency. They had 8–10 Chinese lessons per week, and each lesson lasted for 35 minutes. Among them, there were 129 (82%) Chinese heritage students and 29 (18%) nonheritage students. According to the survey of language use, like nonheritage students, most of the heritage students spoke multiple languages at home, and the highest frequency language was Indonesian (47%), followed by English (29%), Mandarin (11%), Javanese (8%), and others (5%). Their Chinese learning was regarded as foreign language learning. Therefore, the data for heritage and nonheritage students were combined.

### Measures

#### Sentence Dictation in Pinyin

The participants were required to write down the orally presented sentences in Pinyin. There were five sentences altogether, and each sentence was heard twice. For example, participants had to write /tù zi de dù zi è le/ when they heard the Chinese sentence, which means that the rabbit is hungry. All words were from the Chinese textbooks that the participants were familiar with, and we consulted with their Chinese teacher to examine the word appropriateness and tested a few students to ensure that the words have been acquired. The five sentences consisted of 49 syllables, and a total of 16 onsets, 19 rimes and four tones were examined. Onsets included *b p m f d t n l ɡ h j q z zh ch*, and *sh*; rimes incorporated *a e ao an en ang -ɿ -ʅ ia iao iou ian in u uo uei uan ü*, and *üe*; and tones embraced four standard tones (high level, rising, falling-rising, and falling) and a light tone pronounced without its original pitch (e.g., /de/). Reliability was strong (*α* = 0.97). This task is referred to as Pinyin Dictation below.

The scoring standard was 1 point for each correct onset, rime, or tone in 49 syllables, and a maximum score of 147 was possible. The accurate correspondence between Pinyin symbol and sound was the sole criterion of scoring, and we did not pay much attention to the learners’ mastery of the rules for Pinyin orthography, such as word segmentation, serial writing, and capitalizing the first letter in Pinyin sentences. In addition, the participants earned 1 point only for a correct tonal change when needed rather than an original tone. Tonal change means that the tones are altered according to how the adjacent morphemes or words are pronounced. For example, when there are three falling-rising tones side by side (展/zhǎn/览/lǎn/馆/ɡuǎn/), the first two tones change to rising tones automatically (展/zhán/览/lán/馆/ɡuǎn/). Participants were informed of the rule of spelling tone changes in Pinyin sentences before testing.

#### Pinyin Writing for Characters

The participants were required to write out the corresponding Pinyin syllables according to the Chinese characters. A total of 10 characters, all of which were from the Chinese textbooks of participants, included polyphones (e.g., 长 can be pronounced either as /chánɡ/ or as /zhǎnɡ/), homonyms (e.g., 脑 and 恼, both pronounced as /nǎo/), and characters with similar sound and orthography (e.g., 影/yǐnɡ/ and 景/jǐnɡ/). The scoring standard was 1 point for each correct onset, rime, or tone in 10 Pinyin syllables. Thus, there were 30 items for a maximum score of 30 points. Reliability was very good (*α* = 0.89). This task is referred to as Pinyin Tagging below.

#### Character Production

The participants were required to write down the sentences they heard in Chinese characters. For example, participants had to write 足球在桌子上 when they heard the sentence, which means that the football is on the table. There were five sentences altogether, and each sentence was heard twice. We have examined the Chinese textbooks to ensure that the participants already learned the characters selected for the task. There were 32 characters altogether in the five sentences, for a total of 32 points possible. Reliability was very good (*α* = 0.89).

#### Depth of Vocabulary Knowledge

To evaluate the quality of the mental lexicon in participants, we administered two Depth of Vocabulary Knowledge tests. First, the participants were asked to complete a sentence by filling in a blank with one word from a group of words, including distractors with the same morpheme or homophonic morpheme as the correct choice and near-synonyms. For instance, 适合/shìhé/ (suit) and 适应/shìyìnɡ/ (adapt), 适合 is the correct one in the sentence 这条裙子不  你, which means that this skirt does not fit you. There were 10 sentences with blank items, and 1 point was given for the correct completion of one sentence. Second, the participants’ depth of vocabulary knowledge was also measured by asking them to form 10 words with the given 10 morphemes in the task of Pinyin Tagging (e.g., 长 /chánɡ/ 很长; /zhǎnɡ/ 长大), and the participants were allotted 1 point for each correct word. The maximum score was 20. Reliability was very good (*α* = 0.86).

#### Listening Comprehension

Participants’ listening comprehension and reading comprehension were assessed by reference to the Language Ability Self-assessment Scale for intermediate Chinese learners (Wang, 2012). The participants were required to listen and comprehend a short speech, and then judge whether the meaning of a given sentence coincided with the speech. There were altogether eight spoken speeches and eight given sentences; strong interitem reliability has been reported for this task (*α* = 0.93). The participants obtained 1 point for one correct judgment, and the maximum score was 8.

#### Reading Comprehension

Participants were required to read four sentences and two passages and then answer corresponding multiple-choice questions according to the sentences and passages. The questions involved four aspects: specific word or phrase comprehension, attention to detail, text theme and internal relations, information integration, and reasoning. There were 10 questions in total, and reliability was reported to be excellent (*α* = 0.92). The scoring standard was 1 point for one correct question, and the maximum score was 10.

### Data Analysis

Path models were constructed using Mplus8.3 (Muthén and Muthén, 1998-2019) to examine the influence mechanism of Pinyin spelling on reading comprehension. As a first step to testing stringent hypothesis-driven models, an initial model was established in which all possible paths between exogenous and endogenous variables were included, thus a saturated model involving both direct and indirect paths from Pinyin spelling to reading comprehension was created. The saturated model will always provide the best fit to the data because it represents the complete covariance matrix. Hence, the alternative models based on our hypotheses were then tested in which the contribution of Pinyin dictation and Pinyin tagging was only manifested through their influence on concurrent listening comprehension and depth of vocabulary knowledge.

The goodness of fit for the proposed models was evaluated using the chi-square statistic (the *χ*^2^ value), the comparative fit index (CFI), the Tucker-Lewis index (TLI), the root-mean-square error of approximation (RMSEA), and the standardized root-mean-square residual (SRMR). Values that reflect a good fit are as follows: a nonsignificant *χ*^2^ value and CFI and TLI values above 0.95, respectively; and values below 0.06 on the RMSEA and values below 0.08 on the SRMR (Browne and Cudeck, 1993; Hu and Bentler, 1999; Little, 2013; Kline, 2015). Model fit indices were calculated using maximum likelihood estimator (MLM) because it is robust to variations in normality. Nested models were compared using both scaled difference in Chi-squares and Bayesian information criterion (BIC). The chi-square difference test statistic was obtained using hand calculations on regular output of path analysis in Mplus, and the calculation method is available in the research of Satorra and Bentler (2010). The model with a smaller BIC is more acceptable (Raftery, 1996).

## Results

### Preliminary Analyses

Descriptive statistics for all measures are provided in Table 1. For the convenience of comparison, the accuracy rate of each student was also calculated, and all measures were higher than the random level. Noteworthy is that the performance on Chinese tones was comparatively poor in both Pinyin spelling tasks, according to the raw scores of Pinyin Dictation and Pinyin Tagging.

**Table 1:** Descriptive statistics (*N* = 158).

Variable	Mean	SD	Min	Max
Pinyin dictation (max. 147)	102.66	23.22	20	142
Onsets (max. 49)	36.78	8.26	8	49
Rimes (max. 49)	38.11	7.54	8	49
Tones (max. 49)	27.77	9.99	4	47
Pinyin tagging (max. 30)	22.11	5.87	3	30
Onsets (max. 10)	8.25	1.98	1	10
Rimes (max. 10)	8.54	1.88	2	10
Tones (max. 10)	5.31	2.61	0	10
Character production (max. 32)	24.15	5.40	0	32
Depth of vocabulary knowledge (max. 20)	12.96	4.47	0	20
Listening comprehension (max. 8)	6.65	1.33	2	8
Reading comprehension (max. 10)	6.39	2.66	0	10

Min, minimum; Max, maximum.

Table 2 presents the correlation matrix of the variables to be used in the path modeling. Bivariate correlation analyses showed that all of the correlations between Reading Comprehension and the other variables were moderately or highly positive and statistically significant (*r* = 0.40 ~ 0.68). Among them, Depth of Vocabulary Knowledge had the strongest correlation with Reading Comprehension (*r* = 0.68), and it also had strong correlations (*r* = 0.60 ~ 0.69) with the three variables (Pinyin Dictation, Pinyin Tagging, and Character Production), and there were significant correlations among the three variables (*r* = 0.46 ~ 0.70).

**Table 2:** Correlations among all variables.

	1	2	3	4	5	6
1. Pinyin dictation	1					
2. Pinyin tagging	0.46^***^	1				
3. Character production	0.70^***^	0.52^***^	1			
4. Depth of vocabulary knowledge	0.64^***^	0.60^***^	0.69^***^	1		
5. Listening comprehension	0.46^***^	0.26^***^	0.41^***^	0.48^***^	1	
6. Reading comprehension	0.48^***^	0.40^***^	0.49^***^	0.68^***^	0.44^***^	1

*** *p* < 0.001.

### Testing of Path Models

The relations among the variables were further examined by path analysis in structural equation modeling (SEM). Given the established role of Pinyin Dictation, Pinyin Tagging, and Character Production in explaining Chinese reading abilities, these variables were modeled as exogenous, with causal paths to reading related variables. To evaluate the current hypothesis that the Depth of Vocabulary Knowledge and Listening Comprehension mediated the relationship between Pinyin spelling and reading comprehension, these two variables were treated both as endogenous (being predicted from the above three variables) and exogenous in predicting Reading Comprehension.

We first tested the saturated model with all possible parameters freely estimated, such that degrees of freedom are equal to zero and the model fit is perfect. Results showed that path coefficients of Depth of Vocabulary Knowledge (*β* = 0.60, *p* < 0.001) and Listening Comprehension (*β* = 0.14, *p* < 0.05) on Reading Comprehension were significant, while the direct effects of Pinyin Dictation (*β* = 0.03), Pinyin Tagging (*β* = −0.01), and Character Production (*β* = 0.01) on Reading Comprehension were nonsignificant.

**Figure 1:**
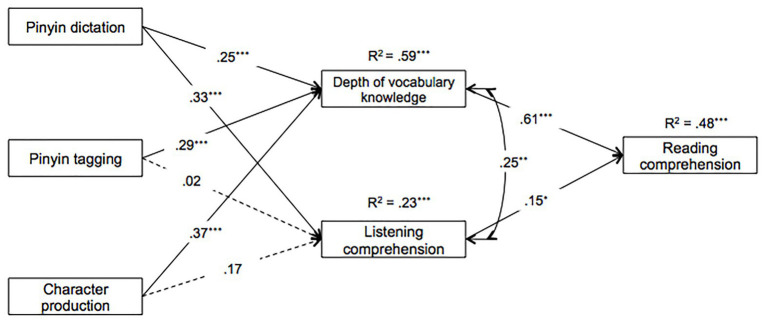
Path model for the relationship between Pinyin spelling and Reading Comprehension, including Depth of Vocabulary Knowledge and Listening Comprehension as two possible mediators (standardized estimates). Solid lines indicate significant paths and dashed lines indicate nonsignificant paths (*p < 0.05; **p < 0.01; ***p < 0.001).

A hypothesis-driven model was then tested by deleting the nonsignificant direct paths from Pinyin Dictation, Pinyin Tagging, and Character Production, which is summarized schematically in Figure 1. Standardized path weights are shown which represent the direct effects, along with the amount of variance accounted for in the endogenous variables (59% for Depth of Vocabulary Knowledge, 23% for Listening Comprehension, and 48% for Reading Comprehension). In the interest of clarity, correlations among exogenous variables are not shown (see Table 2), and coefficients for indirect paths are shown in Table 3. The model as depicted reflected that the influence of Pinyin Dictation was mediated by Depth of Vocabulary Knowledge and Listening Comprehension, and the influence of Pinyin Tagging and Character Production was mediated only by Depth of Vocabulary Knowledge. The results suggested that the model fit was excellent, *χ*^2^(3) = 0.19, *p* = 0.98, CFI = 1, TLI = 1, RMSEA = 0 (90% CI = 0.00–0.00), SRMR = 0.01, and BIC decreased by 14.81 in comparison with the BIC in the saturated model, thus this model was not significantly different from the saturated model, but it was more parsimonious.

**Table 3:** Specific and total indirect effects (standardized).

	Pinyin dictation	Pinyin tagging	Character production
Specific indirect effect 1	0.15	0.18	0.23
Specific indirect effect 2	0.05	0.00	0.02
Total indirect effect	0.20	0.18	0.25

Specific indirect effect 1: the effect mediated by Depth of Vocabulary Knowledge; Specific indirect effect 2: the effect mediated by Listening Comprehension.

A more parsimonious model was obtained by further pruning nonsignificant paths (i.e., the only direct predictor of Listening Comprehension was Pinyin Dictation), and this model provided a more stringent test of our hypothesis. All model indices also indicated an excellent model fit: *χ*^2^(5) = 3.35, *p* = 0.65, CFI = 1, TLI = 1, RMSEA = 0 (90% CI = 0.00–0.09), SRMR = 0.03, and BIC decreased by 6.85 in comparison with the BIC in the last model. The Satorra-Bentler scaled chi-square difference test (*TRd* = 3.26, *df* = 2, and *p* > 0.1) showed that this model was not significantly different from the model in Figure 1.

## Discussion

The present study underscores the importance of Chinese Pinyin skills for Chinese reading abilities in CFL learners. Learning to read Chinese is a cognitively demanding task, because Chinese characters with complex visual-spatial configurations and phonological opacity are difficult to recognize. Universal phonological principle (Perfetti et al., 1992; Perfetti and Tan, 1998) demonstrates that written Chinese, similar to written English, involves phonological activation and processing (Perfetti et al., 2005). Our results have confirmed that Pinyin spelling skills have an effect on Chinese reading comprehension in CFL learners. Indeed, Pinyin spelling skills likely boost reading development by at least two pathways among CFL learners.

In line with our first hypothesis, Pinyin dictation had an indirect effect on reading comprehension that was mediated by listening comprehension. As mentioned above, Pinyin dictation actually involves a phonological encoding process of Chinese speech. Successful performance of Pinyin encoding indicates that CFL learners have not only categorized fine-grained Chinese phonological representations to perceive Chinese phonemes as distinct from similar segments in L1 or Chinese but also well interpreted and retained Chinese speech information, including words and grammar. Thus, Pinyin encoding requires more than Pinyin knowledge and writing ability, and it is, in effect, a comprehensive task to analyze spoken Chinese by using explicit Pinyin representations. To some extent, listening comprehension refers to a wide spectrum of oral language proficiency, and Pinyin as a physical entity of spoken Chinese plays an irreplaceable role in oral vocabulary learning and oral communication, which may be the more general reason why Pinyin encoding skill affects reading comprehension through the mediating effect of listening comprehension.

It is interesting to compare the predictive power of Pinyin dictation and character production on listening comprehension. One can conclude that both Pinyin dictation and character production tasks are first listening tasks, and the only difference between them lies in the encoding symbols used to analyze and spell Chinese, after we have controlled that the words used in both tasks were familiar to participants. However, only Pinyin dictation had a significant predictive effect on listening comprehension (*β* = 0.33, *p* < 0.001), which suggests that Pinyin dictation did capture something that could not be measured by character production. Only phonological symbols, not opaque characters, can influence the ability to comprehend Chinese spoken language. Beyond that, the reason Pinyin tagging could not predict listening comprehension may be that the task assessed the ability to access and produce the phonological representation through orthographic input, which had no direct relationship with listening comprehension.

The findings also confirmed our second prediction that Pinyin spelling skills (including Pinyin dictation and Pinyin tagging) had an indirect effect on reading comprehension that was mediated by the depth of vocabulary knowledge, even after controlling for the effect of character production on reading comprehension. The two spelling tasks reflected two facets of the role in Pinyin, encoding oral Chinese and transcoding between Chinese characters and Pinyin forms. Pinyin encoding is related to a direct manipulation of spoken phonological information. Pinyin transcoding demands one to decode Chinese characters accurately first and then encode phonological information in Pinyin. Accordingly, both spelling skills can facilitate specifying the phonological representations of words, and thus affect the performance of reading comprehension. In addition, Pinyin transcoding can also assess the compact degree of orthography, phonology, and semantics in Chinese characters, and reading comprehension is what USES the above bond to decode written words and access the meaning of words (Dong et al., 2012).

Our results not only supported the universal phonological principle in reading but also are consistent with numerous previous studies by showing the strong predictive effect of vocabulary knowledge on reading comprehension (*β* = 0.61, *p* < 0.001; Share, 2008; Verhoeven et al., 2019). Compared with listening comprehension (*β* = 0.15, *p* < 0.05), the depth of vocabulary knowledge played a more important role in predicting reading comprehension, which may be accounted for by the Reading Systems Framework (Perfetti and Stafura, 2014). The framework assumes that the lexicon sits astride two reading systems: one is the word identification system and the other is the comprehension system, and the lexicon plays the linking role such as integrating a word with the ongoing representation of the text (Perfetti and Hart, 2001; Perfetti, 2007). Hence, the depth of vocabulary knowledge rather than listening comprehension was the main mediator of the relationship between Pinyin spelling and reading comprehension.

The long-term and multifaceted influence of Pinyin on CFL abilities in this study is worth further discussion. The participants in the study were senior primary students, and a long time had passed since their concentrated education of Pinyin in Grade 1. Nevertheless, Pinyin spelling skills can still affect many Chinese language abilities, including listening comprehension, vocabulary knowledge, and reading abilities. We believe that there might be two explanations for the contribution of Pinyin. First, Pinyin can facilitate basic and underlying phonological processing that higher-level CFL/CSL abilities rely on. For native Chinese children, who have acquired the phonological system unconsciously and reached a certain level of oral ability, learning the letter-sound correspondence in Pinyin can further strengthen their phonological awareness (Shu et al., 2008; Lin et al., 2010; Pan et al., 2011; Li et al., 2012; Chen et al., 2016). However, for CFL/CSL learners who have never been exposed to Chinese, Pinyin is actually the concretization and specification of their Chinese phonological representations, and they develop Chinese phonological awareness based on the Pinyin encoding system. The influence of Pinyin on the metalinguistic awareness in CFL/CSL learners lays a foundation for the lasting and full impact of Pinyin on higher-level Chinese abilities.

Second, the phonological opacity of Chinese characters contributes to the positive relationship between Pinyin skills and higher-level Chinese abilities of CFL/CSL learners. For example, reading comprehension depends on successful word reading, and word decoding is an important part of word reading (Perfetti, 2007). However, the impact of word decoding skills was found to be modulated by the transparency of the orthography, being stronger and tending to remain for a longer period in languages with opaque orthography (Florit and Cain, 2011; Joshi et al., 2015; Wong, 2016). That is, the ability to retrieve phonological information from Chinese characters that have been historically viewed as meaning-based rather than speech-based develops at a slower pace than in alphabetic languages. Consequently, the Pinyin spelling skills that help word decoding in this study will remain highly influential on learners’ reading performances *via* the depth of vocabulary knowledge.

## Practical Implications and Limitations

The results of the study have to some degree discredited the views that Pinyin may interfere with the acquisition of native words in children with an alphabet language as L1, or they can spell Pinyin without learning. Chinese Pinyin uses internationally accepted Roman letters, and the orthographic similarities between Pinyin and alphabetic writing system of L1 may facilitate children’s biliteracy development (Geva and Siegel, 2000; Xu and Liu, 2016). In the process of comparing and distinguishing the same letters with different sounds, children’s orthographic awareness, metalinguistic abilities, and cognitive control abilities can also be further improved. In addition, there were still some difficulties relating lexical tones (with only 59.08 percent accurate on the lexical tones of Pinyin Dictation task) and Chinese coronal affricates and fricatives (with less than 80 percent accurate on the *z zh ch q* of Pinyin Dictation task) for the learners with intermediate Chinese proficiency in this study. Furthermore, Chinese learners who have never learned Pinyin do not include Pinyin as a phonetic symbol in their phonological representations, and they have no mental images to refer to when they mentally perceive and operate phonetic information. The mental image of phonetic symbolization can facilitate the processing and transmission of phonetic information (Ehri et al., 1984). Thus, compared with learners who have learned Pinyin systematically, CFL/CSL learners who have never been exposed to Pinyin are likely to perform worse in tasks involving Chinese phonological processing, such as oral vocabulary and reading ability. Therefore, systematic Pinyin instruction is essential.

The findings of this study have demonstrated the importance of Pinyin skills for CFL proficiency, from which at least two practical implications can be concluded. First, the long-term and multifaceted influence of Pinyin spelling not only reinforces the indispensability of Pinyin in CFL/CSL learning but also indicates that the Pinyin instruction should be carried on to a more in-depth degree, leaving a reasonable time window for Pinyin to make a significant contribution to further growth in CFL/CSL abilities. In addition, learners rely on visual Pinyin encoding symbols to acquire the Chinese phonological system and achieve fine-grained phonological representations; thus CFL/CSL teachers are supposed to lay stress on the distinctiveness of some specific phonetic contrasts, including the tones in Chinese that are difficult for perception and production in learners. After mastering the Pinyin encoding system within a short time, learners can ultimately make big gains with the small skillset.

Second, it is necessary to differentiate between Pinyin teaching and Pinyin learning or practice. The former lasts for a short time and mainly focuses on learning the Scheme of the Chinese Phonetic Alphabet, including Pinyin letters and spelling rules. The latter is long-term for the purpose of developing all-round Chinese abilities and involves the use of Pinyin to consolidate and acquire Chinese vocabulary and sentence patterns, as well as foster oral communication. After distinguishing the two theoretically, CFL/CSL teachers can link up Pinyin and Chinese character teaching and improve CFL/CSL teaching efficiency. Furthermore, Pinyin learning or practice should center around the two cognitive functions of Pinyin, encoding spoken Chinese and transcoding Chinese characters in Pinyin forms.

Several limitations to this study need to be recognized. First, when measuring the listening comprehension abilities of learners, sentences that needed to be judged as true or false were presented in the form of Chinese characters rather than orally presented, which may influence the effects of listening comprehension in this paper but it also showed the role of Pinyin spelling from the opposite side. Second, the paper has shown the long-term effects of Pinyin spelling on reading comprehension. However, admittedly, definite evidence for such a causal connection would require a longitudinal experimental showing that early Pinyin spelling skills facilitate later Chinese reading comprehension.

The present study expands our understanding of the cognitive functions in Pinyin by exploring how senior primary CFL students’ Pinyin spelling skills, *via* Pinyin dictation and Pinyin tagging, are associated with Chinese reading abilities. The results indicated that the contribution of Pinyin spelling was manifested through its influence on listening comprehension and depth of vocabulary knowledge. The long-term and multifaceted influence of Pinyin on CFL/CSL abilities warrants further investigation.

## Data Availability Statement

The raw data supporting the conclusions of this article will be made available by the authors, without undue reservation.

## Ethics Statement

The studies involving human participants were reviewed and approved by Xin Zhong School in Surabaya, Indonesia. Written informed consent to participate in this study was provided by the participants’ legal guardian/next of kin.

## Author Contributions

CX designed the study. HR performed the study and collected the data. HX analyzed the data and prepared the draft. CX and HX contributed to the final manuscript writing. All authors contributed to the article and approved the submitted version.

### Conflict of Interest

The authors declare that the research was conducted in the absence of any commercial or financial relationships that could be construed as a potential conflict of interest.

## References

[ref1] Best C. T. Tyler M. D. (2007). “Nonnative and second-language speech perception: commonalities and complementarities” in Language experience in second language speech learning. eds. Bohn O. Munro M. J. (Amsterdam Philadelphia: John Benjamins Publishing Company), 13–35.

[ref2] Bi Y. Han Z. Zhang Y. (2009). Reading does not depend on writing, even in Chinese. Neuropsychologia 47, 1193–1199. 10.1016/j.neuropsychologia.2008.11.006, PMID: 19056407

[ref3] Bradley L. (1988). Making connections in learning to read and spell. Appl. Cogn. Psychol. 2, 3–18. 10.1002/acp.2350020103

[ref4] Browne M. W. Cudeck R. (1993). “Alternative ways in assessing model fit” in Testing structural equation models. eds. Bollen K. Long J. S. (Newbury Park, CA: Sage), 136–162.

[ref5] Bryant P. E. Bradley L. (1980). “Why children sometimes write words which they do not read” in Cognitive processes in spelling. ed. Frith U. (New York: Academic Press), 355–370.

[ref6] Chen L. Zhou X. Wang Y. (2016). The effectiveness and mechanism of pinyin in Chinese learning. Stud. Psychol. Behav. 5, 715–720.

[ref7] Cheng M. Zhao J. (1985). Some problems in basic Chinese phonology teaching. The world Chinese language teaching association. The first international Chinese language teaching symposium selected papers, 225–234.

[ref8] Chung K. H. (2002). Effective use of Hanyu pinyin and English translations as extra stimulus prompts on learning of Chinese characters. Educ. Psychol. 22, 149–164. 10.1080/01443410120115238

[ref9] Deng D. (2018). Production and perception of Chinese coronal affricates and fricatives by native Korean speakers. Chinese Teaching in the World. 32, 110–125.

[ref10] Ding Y. Liu R. -D. McBride C. A. Fan C. -H. Xu L. Wang J. (2018). Pinyin and English invented spelling in Chinese-speaking students who speak English as a second language. J. Psycholinguist. Res. 45, 1163–1187. 10.1007/s10936-018-9585-4, PMID: 29736593

[ref11] Dong Q. Li H. Wu X. Pan J. Zhang Y. Ruan S. (2012). Reading-related cognitive skills deficits in children with Chinese developmental dyslexia. Chin. J. Clin. Psychol. 20, 798–801. 10.16128/j.cnki.1005-3611.2012.06.025

[ref12] Droop M. Verhoeven L. (2003). Language proficiency and reading ability in first-and second-language learners. Read. Res. Q. 38, 78–103. 10.1598/RRQ.38.1.4

[ref13] Ehri L. (1989). The development of spelling knowledge and its role in reading acquisition and reading disabilities. J. Learn. Disabil. 22, 356–365. 10.1177/002221948902200606, PMID: 2738469

[ref14] Ehri L. Deffner N. Wilce L. (1984). Pictorial mnemonics for phonics. J. Educ. Psychol. 76, 880–893. 10.1037/0022-0663.76.5.880

[ref15] Flege J. E. (1999). “The relation between L2 production and perception” in The proceedings of the 14th ICPhS. San Francisco; August 1-7, 1999; 1273–1276.

[ref16] Florit E. Cain K. (2011). The simple view of reading: is it valid for different types of alphabetic orthographies? Educ. Psychol. Rev. 23, 553–576. 10.1007/s10648-011-9175-6

[ref17] Geva E. Siegel L. S. (2000). Orthographic and cognitive factors in the concurrent development of basic reading skills in two languages. Read. Writ. 12, 1–30. 10.1023/A:1008017710115

[ref18] Hanley R. (2005). “Learning to read Chinese” in The science of reading: A handbook. eds. Snowling M. J. Hulme C. (London, UK: Blackwell), 316–335.

[ref19] Hoover W. A. Gough P. B. (1990). The simple view of reading. Read. Writ. 2, 127–160. 10.1007/BF00401799

[ref20] Hu L. Bentler P. M. (1999). Cutoff criteria for fit indices in covariance structure analysis: conventional criteria versus new alternatives. Struct. Equ. Model. 6, 1–55. 10.1080/10705519909540118

[ref21] Institute of Linguistics, Chinese Academy of Social Sciences (2004). Xinhua dictionary. 10th Edn. Beijing, China: Commercial Press.

[ref22] Janssen C. Segers E. McQueen J. M. Verhoeven L. (2017). Transfer from implicit to explicit phonological abilities in first and second language learners. Biling. Lang. Cogn. 20, 795–812. 10.1017/S1366728916000523

[ref23] Joshi R. M. Ji X. Breznitz Z. Amiel M. Yulia A. (2015). Validation of the simple view of reading in Hebrew—a semitic language. Sci. Stud. Read. 19, 243–252. 10.1080/10888438.2015.1010117

[ref24] Kline R. B. (2015). Principles and practice of structural equation modeling. 4th Edn. New York, NY: Guilford Press.

[ref25] Li H. Shu H. McBride-Chang C. Liu H. Peng H. (2012). Chinese children’s character recognition: visuo-orthographic, phonological processing and morphological skills. J. Res. Read. 35, 287–307. 10.1111/j.1467-9817.2010.01460.x

[ref26] Lin D. McBride-Chang C. Shu H. Zhang Y. P. Li H. Zhang J.. (2010). Small wins big: analytic pinyin skills promote Chinese word reading. Psychol. Sci. 21, 1117–1122. 10.1177/0956797610375447, PMID: 20581343

[ref27] Little T. D. (2013). Longitudinal structural equation modeling. New York/London: The Guilford Press.

[ref28] Lu J. (2013). On the scheme of Chinese phonetic alphabet and Chinese teaching. Appl. Linguist. 11–14.

[ref29] Ma S. Zhang X. Hatfield H. Wei W. H. (2020). Pinyin is an effective proxy for early screening for mandarin-speaking children at risk of reading disorders. Front. Psychol. 11:327. 10.3389/fpsyg.2020.00327, PMID: 32174873 PMC7055296

[ref30] Mattingly I. G. (1992). “Linguistic awareness and orthographic form” in Orthography, phonology, morphology, and meaning. eds. Frost R. Katz L. (Amsterdam: Elsevier Science), 11–26.

[ref31] McBride-Chang C. (1998). The development of invented spelling. Early Educ. Dev. 9, 147–160. 10.1207/s15566935eed0902_3

[ref32] Muthén L. Muthén B. (1998-2019). Mplus user’s guide. Los Angeles, CA: Muthén & Muthén.

[ref33] Nation I. S. P. (2001). Learning vocabulary in another language. Cambridge: Cambridge University Press.

[ref34] Ouellette G. Sénéchal M. (2008). Pathways to literacy: a study of invented spelling and its role in learning to read. Child Dev. 79, 899–913. 10.1111/j.1467-8624.2008.01166.x, PMID: 18717897

[ref35] Pan J. McBride-Chang C. Shu H. Lin H. Zhang Y. Li H. (2011). What is in the naming? A 5-year longitudinal study of early rapid naming and phonological sensitivity in relation to subsequent reading skills in both native Chinese and English as a second language. J. Educ. Psychol. 103, 897–908. 10.1037/a0024344

[ref36] Perfetti C. A. (2007). Reading ability: lexical quality to comprehension. Sci. Stud. Read. 11, 357–383. 10.1080/10888430701530730

[ref37] Perfetti C. A. Hart L. (2001). “The lexical quality hypothesis” in Precursors of functional literacy. eds. Verhoeven L. Elbro C. Reitsma P. (John Benjamins: Amsterdam/Philadelphia), 189–214.

[ref38] Perfetti C. A. Liu Y. Tan L. H. (2005). The lexical constituency model: some implications of research on Chinese for general theories of reading. Psychol. Rev. 112, 43–59. 10.1037/0033-295X.112.1.43, PMID: 15631587

[ref39] Perfetti C. A. Stafura J. (2014). Word knowledge in a theory of reading comprehension. Sci. Stud. Read. 18, 22–37. 10.1080/10888438.2013.827687

[ref40] Perfetti C. A. Tan L. H. (1998). The time course of graphic, phonological, and semantic activation in visual Chinese character identification. J. Exp. Psychol. Learn. Mem. Cogn. 24, 101–118. 10.1037/0278-7393.24.1.101

[ref41] Perfetti C. A. Zhang S. Berent I. (1992). “Reading in English and Chinese: evidence for a “universal” phonological principle” in Orthography, phonology, morphology, and meaning. eds. Frost R. Katz L. (Amsterdam: North-Holland), 227–248.

[ref42] Raftery A. E. (1996). “Bayesian model selection in social research (with discussion)” in Sociological methodology. Vol. 25. ed. Marsden P. V. (Oxford: Basil Blackwell), 111–163.

[ref43] Read C. (1971). Pre-school children’s knowledge of English phonology. Harv. Educ. Rev. 41, 1–34. 10.17763/haer.41.1.91367v0h80051573

[ref44] Read J. (1993). The development of a new measure of L2 vocabulary knowledge. Lang. Test. 10, 355–371. 10.1177/026553229301000308

[ref45] Satorra A. Bentler P. M. (2010). Ensuring positiveness of the scaled difference chi-square test statistic. Psychometrika 75, 243–248. 10.1007/s11336-009-9135-y, PMID: 20640194 PMC2905175

[ref46] Share D. L. (2008). On the anglocentricities of current reading research and practice: the perils of overreliance on an “outlier” orthography. Psychol. Bull. 134, 584–615. 10.1037/0033-2909.134.4.584, PMID: 18605821

[ref47] Shu H. Liu B. X. (1994). Effects of pinyin on early Chinese reading of lower graders. Psychol. Dev. Educ. 3, 11–15.

[ref48] Shu H. Peng H. McBride-Chang C. (2008). Phonological awareness in young Chinese children. Dev. Sci. 11, 171–181. 10.1111/j.1467-7687.2007.00654.x, PMID: 18171377

[ref49] Siok W. T. Fletcher P. (2001). The role of phonological awareness and visual-orthographic skills in Chinese reading acquisition. Dev. Psychol. 37, 886–899. 10.1037/0012-1649.37.6.886, PMID: 11699761

[ref50] Snow C. E. (2002). Reading for understanding: Toward a R&D program in reading comprehension. Santa Monica, CA: Rand.

[ref51] Tan L. H. Spinks J. A. Eden G. F. Perfetti C. A. Siok W. T. (2005). Reading depends on writing, in Chinese. Proc. Natl. Acad. Sci. U. S. A. 102, 8781–8785. 10.1073/pnas.0503523102, PMID: 15939871 PMC1150863

[ref52] Verhoeven L. Voeten M. Vermeer A. (2019). Beyond the simple view of early first and second language reading: the impact of lexical quality. J. Neurolinguist. 50, 28–36. 10.1016/j.jneuroling.2018.03.002

[ref53] Wan Y. (2012). The role of pinyin transcription and Chinese characters and their relationship in teaching Chinese as a foreign language. Chinese Teaching in the World. 26, 409–418.

[ref54] Wang J. (2012). Compilation and testing of the language proficiency self-rating scale for intermediate Chinese learners. China Exam. 11, 11–16. 10.19360/j.cnki.11-3303/g4.2012.11.002

[ref55] Wang L. (2013). The theoretical explanation of "The Plan of Chinese Phonetic Alphabet" and the basic concepts of teaching Chinese phonetic alphabet. Appl. Linguist. 7–11. 10.16499/j.cnki.1003-5397.2013.04.004

[ref57] Wang Y. McBride C. (2016). Character reading and word reading in Chinese: unique correlates for Chinese kindergarteners. Appl. Psycholinguist. 37, 371–386. 10.1017/S014271641500003X

[ref56] Wang Y. McBride-Chang C. Chan S. (2014). Correlates of Chinese kindergarteners’ word reading and writing: the unique role of copying skills? Read. Writ. 27, 1281–1302. 10.1007/s11145-013-9486-8

[ref59] Wong Y. K. (2016). Relationships between reading comprehension and its components in young Chinese-as-a-second-language learners. Read. Writ. 30, 969–988. 10.1007/s11145-016-9708-y

[ref60] Xu C. Liu J. (2016). A brief discussion on Chinese pinyin and Chinese teaching for overseas children. J. Yunnan Normal Univ. (Teach Res CFL) 14, 1–6. 10.16802/j.cnki.ynsddw.2016.05.002

[ref61] Yin L. Li W. Chen X. Anderson R. C. Zhang J. Shu H. (2011). The role of tone awareness and pinyin knowledge in Chinese reading. Writ. Syst. Res. 3, 59–68. 10.1093/wsr/wsr010

[ref62] Zhang S. Z. Georgiou G. K. Inoue T. Zhong W. Shu H. (2020). Do pinyin and character recognition help each other grow? Early Child. Res. Q. 53, 476–483. 10.1016/j.ecresq.2020.06.004

